# Educating for Responsible Research Practice in Biomedical Sciences: Towards Learning Goals

**DOI:** 10.1007/s11191-021-00295-y

**Published:** 2021-11-01

**Authors:** Elianne M. Gerrits, Annelien L. Bredenoord, Marc H. W. van Mil

**Affiliations:** 1 Center of Education and Training, University Medical Center Utrecht, Utrecht University, Utrecht, The Netherlands; 2 School of Philosophy, Erasmus University Rotterdam, Rotterdam, The Netherlands; 3 Center for Molecular Medicine, University Medical Center Utrecht, Utrecht University, Utrecht, The Netherlands

## Abstract

New developments in the field of biomedicine can have extensive implications for society. To steer research efforts in a responsible direction, biomedical scientists should contribute to a forward-looking ethical, and societal evaluation of new developments. However, the question remains how to equip students sufficiently with the skills they need to contribute to this evaluation. In this paper, we examine how the four dimensions of Responsible Research and Innovation (anticipation, reflexivity, inclusivity, and responsiveness) inform the identification of learning goals and teaching approaches that contribute to developing these skills in biomedical scientists. We suggest that these educational approaches focus on the skills to anticipate intended and unintended outcomes, reflect on the epistemological and moral aspects of research practice, and be inclusive of the variety of voices in society. We argue that if these dimensions are properly integrated into biomedical curricula, they will help students develop the attitudinal aspects necessary for becoming responsive, and prepare them for implementing the dimensions of responsible research into their daily practice. This paper focuses specifically on skills biomedical scientists need for the responsible conduct of research. Therefore, our analysis results, at least in part, in domain-specific recommendations. We invite educators from other disciplines to do the same exercise, as we believe this could lead to tailored educational approaches by which students from various disciplinary backgrounds learn how they each have a role in contributing to socially robust and morally responsible research practice.

## Introduction

Scientific developments come with increasingly large ethical and societal implications that require careful consideration. For science education, this means that curricula, especially in higher education, should not focus only on scientific content but also on preparing students for their role in overseeing and discussing the impact of scientific innovation in society. This is especially true for the field of biomedical sciences, in which new developments like whole-genome analysis and the discovery of CRISPR-cas9 lead to extensive academic and societal debate on safety and desirability. Although there is a widespread pledge that (future) bioscientists should be involved in dealing with the ethical and societal issues in their field (Bosch, 2018; Douglas, 2003; Sugarman & Bredenoord, 2020), the question remains how biomedical curricula can be adjusted to equip students with the necessary skills to consider these ethical and societal dimensions. In other words, what are the learning goals that fit these ambitions and how can these learning goals be brought into practice?

Pledges for more extensive deliberation of the ethical and societal impact of biomedical innovation have not only been made in academia but can also be recognized in several policy programs and initiatives. These include the ethical, legal, and societal issues (ELSI) framework developed alongside the Human Genome Project in 1990 (Langfelder & Juengst, 1993), and the Responsible Research and Innovation (RRI) framework developed in 2010 as part of the European Union program for research and innovation “Horizon 2020” (Von Schomberg, 2013). Analyzing these frameworks, a broad commonality can be recognized that shows a normative consensus on how science should be conducted. This normative consensus can be summarized as steering the processes and outcomes of research and innovation so they are ethically acceptable for society by involving different academic disciplines, stakeholders, and the public upstream in the research process. Implementing such an upstream ethical and societal evaluation of biomedical developments not only asks for the contribution of experts from outside of the biomedical field, but also demands a different way of thinking and acting from biomedical researchers. They should, for instance, be able to recognize research outcomes that could cause ethical conflicts. Additionally, they should be open to collaborating with other stakeholders and academic disciplines early in the research process, and be transparent when communicating about their work to a broader audience. To ensure that future biomedical researchers are prepared to contribute to the proposed vision of ethical and socially responsible research, their educational program must be closely attuned to developing congruent skills. In this paper, we aim to establish what these congruent skills are and define learning goals for future biomedical scientists.

As inspiration and direction for specifying these learning goals, we use previous work by Tassone and colleagues in which Responsible Research and Innovation competencies for students in higher education are described (Tassone & Eppink, 2016; Tassone et al., 2017). These competencies are structured along the four dimensions of RRI, described in work by Stilgoe and colleagues (Stilgoe et al., 2013). These four dimensions, anticipation, reflexivity, inclusivity, and responsiveness, form the main processes that support responsible conduct of research and innovation. By defining competencies based on all four dimensions of RRI, Tassone and colleagues establish a framework of competencies higher education students need to participate in these RRI processes. As their work is meant as a tool to guide educators in (re-)designing their curricula, Tassone and colleagues invite educators to experiment with the suggested framework within their own context. When we follow this suggestion and project these competencies on our specific context of biomedical education, we feel that an additional step has to be made to translate these general competencies into specific learning goals suitable for biomedical students.

In this paper, we determine specific learning goals for biomedical sciences students by starting with a reflection on how each of the four RRI dimensions and their corresponding competencies proposed by Tassone and colleagues fit into a biomedical sciences educational program. We do this in the light of closely related scholarly work in other disciplines (e.g., ethics, social sciences, science education, and moral education). The specific learning goals we identify lay the foundations for a backward design approach in which concrete curriculum innovations follow from well-established curricular endpoints (Wiggins & McTighe, 2005). We organize our learning goals as suggested by Tassone et al., along the lines of the cognitive, affective, and psychomotor domains. Additionally, we identify learning goals with taxonomy descriptors indicating their level of depth, based on the work of Bloom (1956), Krathwohl (2010), Krathwohl et al. (1964), and Dave (1970). This clarifies the position of the learning goals in existing or newly developed learning progressions within the curriculum. Finally, we complement our learning goals with suggestions for concrete teaching approaches that fit these goals. A summary of our findings can be found in Table 1.

**Table 1:** Dimension and competencies of Responsible Research and Innovation, and congruent learning goals for biomedical scientists, organized in the cognitive, affective, and psychomotor domains. The learning goals are supplemented with examples of teaching activities that support that specific learning goal

Dimensions (Stilgoe et al. (2013) and competencies (Tassone et al. (2017)	Learning goals	Learning domain (Bloom (1956), Krathwohl, (2010), Krathwohl et al. (1964) and Dave (1970))	Example of teaching activity
Anticipation *• Future studies competencies* *• Future-oriented competencies* *• Proactivity*	Demonstrate how historic and current data can be used to predict the future impact of biomedical research	Cognitive (apply)	Students compare historic examples of controversial biomedical innovation to current cases to look for similarities and new possibilities to anticipate negative outcomes
	Differentiate between hard and soft impacts of biomedical research outcomes	Cognitive (analyze)	Students list possible implications of biomedical innovations, specifically focusing on soft impacts, which are harder to predict
	Use and appreciate the skill of moral imagination to reflect on the soft impact of biomedical innovation	Affective (value)	Students try to place themselves in a scenario, to reflect on how they would experience the impact of a specific situation
	Deliberate the desirability of future research outcomes	Cognitive (evaluate) Affective (organize)	Students evaluate the benefits and harms of new innovations and use arguments to describe the desirability of these innovations
	Create own scenarios to anticipate research outcomes, taking into account their uncertainties	Cognitive (create) Psychomotor (articulate)	Students create scenario’s in which they describe how implementing a specific innovation in society might lead to societal changes
Reflexivity *• Self-awareness* *• Situational awareness* *• Social awareness/empathy* *• Ethical thinking* *• Disruptive thinking*	Understand how biomedical science produces knowledge	Cognitive (understand) Affective (receive)	Students reflect on how research questions and hypotheses are formulated and when hypotheses can be confirmed or disproved
	Understand the justification of research methodologies	Cognitive (understand) Affective (respond)	Students reflect from a philosophical perspective if the used research approach is adequate for confirming or disproving hypotheses
	Have adequate reasoning skills, recognize and avoid reasoning fallacies	Cognitive (understand) Affective (respond)	Students look at different types of reasoning fallacies and learn how to recognize them
	Understand why science needs reproducibility and evaluate the reproducibility of a research study	Cognitive (application) Affective (value)	Students discuss the pitfalls of reproducibility and how to avoid them
	Recognize the importance of scientific integrity and evaluate the integrity of a research study	Cognitive (application) Affective (value)	Students discuss the pitfalls of scientific integrity and how to avoid them
	Critically reflect on scientific work produced by oneself and others	Cognitive (evaluate)	Students discuss scientific work that was retracted because of research mistakes
	Know and understand the rules regarding the use of animal models in biomedical research	Cognitive (understand) Affective (value)	Students discuss the necessity of animal testing as well as the opinions of animal rights organizations
	Know and understand the rules regarding the use of human subjects	Cognitive (understand) Affective (value)	Students reflect on current and historical examples of research on human subjects and discuss how to avoid exploitation
	Recognize the ethical aspects of a situation	Cognitive (evaluate) Affective (organize)	Students look for patterns in the ethical aspects that can be recognized in several cases of biomedical innovation
	Be able to reason about the ethical aspects in a situation and discuss a justifiable course of action	Psychomotor (articulate)	Students discuss the ethical aspects of a situation from multiple perspectives and try to come to a strong conclusion
Inclusivity *• Trans-disciplinary collaboration* *• Participatory ability* *• Multi-perspective and intercultural communication* *• Openness and transparency*	Become aware of one’s disciplinary perspective	Affective (receive)	Two teachers, one with a biomedical background, one with a different disciplinary background, together discuss a biomedical topic, both from their own disciplinary perspective
	Recognize and value different disciplinary perspectives in multi-disciplinary collaborations	Cognitive (analyze) Affective (value)	Two teachers, one with a biomedical background, one with a different disciplinary background, discuss a biomedical topic, both from their own disciplinary perspective
	Understand the epistemological foundations of different disciplines	Cognitive (understand)	Students look at research papers from different disciplinary journals, and specifically at the research questions and methodologies used
	Value emotional perspectives in inclusive deliberation with different stakeholders and members of the public	Affective (value)	Students participate in a role-playing exercise in which students take a specific emotional perspective
	Communicate scientific knowledge to people with different emotional perspectives and levels of scientific knowledge	Psychomotor (perfect)	Students participate in dialogues with people from different social and cultural backgrounds
	Listen and learn while participating in bi-directional forms of communication	Affective (internalize) Psychomotor (embody)	Students formulate new biomedical research questions together with members from the public
	Be sensitive in communication about uncertainty and risk in service of open and transparent communication	Affective (internalize) Psychomotor (embody)	Students practice translating research findings into material (e.g., a news article or a video) for a layperson audience. This can concern both published research findings, as well as experiments the students did themselves
Responsiveness: *• Navigate complexity/wickedness* *• Agency* *• Adaptability*	Recognize the role of biomedical research in creating and solving complex/wicked problems	Cognitive (analyze)	Students analyze a complex problem like sustainability so they will appreciate that biomedical research has a role in both creating and solving these complex problems
	Use interdisciplinary competencies to integrate knowledge from different fields	Psychomotor (embody)	Students collaborate in multidisciplinary teams to solve complex problems that cross-disciplinary boundaries
	Have confidence in one’s competencies of anticipation, reflexivity, and inclusivity	Psychomotor (embody)	Students participate in reflective sessions with fellow students as part of their internships. In these sessions, students reflect on how the dimensions of anticipation, reflexivity, and inclusivity are integrated in their projects

## Learning Goals in Four Dimensions

### Anticipation

The first dimension described by Stilgoe and colleagues is anticipation. It is described as improving foresight by systematic thinking and asking “what if…?” questions to predict and possibly redirect research outcomes (Stilgoe et al., 2013). For a proper evaluation of the societal impact of biomedical developments, ethical and societal aspects must be considered in parallel, meaning that they should be discussed alongside the development process. This parallel evaluation makes it possible to guide and redirect research efforts during the process, rather than solely providing a hindsight assessment. To make sure this evaluation is comprehensive, experts from different disciplines (e.g., ethicists, social scientists, and legal experts) have to work in close collaboration with biomedical scientists (Jongsma & Bredenoord, 2020). However, to make these collaborations successful, biomedical scientists need competencies that are not usually part of their educational program.

Tassone and colleagues identified future studies competencies, future-oriented ethical competencies, and proactivity as the competencies needed for anticipation (Tassone et al., 2017). These competencies highlight the importance of learning how an educated and empirically grounded speculation of the future can be made in which ethical aspects are taken into consideration. Proactivity can be seen as understanding that early consideration of possible future outcomes is important for steering research processes into a desirable direction. An often-used method for thinking about the future is the use of scenarios, as they stimulate active engagement in consideration of future outcomes (Vervoort et al., 2015). We, therefore, argue that using scenarios in education could be a useful approach when teaching students how to anticipate the outcomes of biomedical innovation.

#### Future Scenarios to Support Anticipation

Scenarios for studying the future should not be based on complete speculation but should follow an educated imagination. Therefore, scenarios can be seen as empirically grounded speculations on the technical possibilities of new developments based on current and historical information (Lucivero, 2016; Stemerding et al., 2010). By engaging in empirically grounded scenarios, science students may become aware of the translational capacity of historical events and current data for anticipating the future and how certain choices can shape future outcomes.

However, future-oriented scenarios should not only anticipate the technical possibilities but also the effects technical developments could have on individuals and society (Stemerding et al., 2010; Vervoort et al., 2015). To thoroughly understand the impact new developments can have on society, it is useful for students to be aware of the difference between hard and soft impacts (Assen et al., 2021; Boerwinkel et al., 2014). Hard impacts include effects such as health risks, costs, and environmental effects. These effects are quantifiable and result in concrete outcomes that can be marked as desirable or undesirable. However, solely focusing on hard impacts does not provide a complete overview of the possible ethical and societal implications, and soft impacts should also be considered. Soft impacts are the effects that new developments can have on behavior, societal norms, and values. These aspects are more difficult to quantify, and therefore increase the level of uncertainty when predicting the impact of innovation (Boenink et al., 2010). With the inclusion of soft impacts in future scenarios, it becomes more complicated to evaluate the desirability of the described situation.

To assist in reflecting on the soft impact in scenarios and the soft impact of biomedical innovation, the skill of moral imagination can be used. Individuals use their moral imagination continuously when making ethical decisions by imagining the effects of a decision. When doing this, we place ourselves in that future and imagine how that experience would be for us (Coeckelbergh, 2007). Although people use moral imagination daily, it is not a skill that researchers often explicitly use in their work. Asking students to imagine how they would feel in a described future brought about by certain research outcomes might help them become aware of the value of moral imagination in the evaluation of biomedical research. Seeing the value of the skill of moral imagination to evaluate new biomedical innovations requires students to become aware that they have this skill, and become devoted to using this skill to consider the ethical and societal dimensions of their research. After students become aware that their moral imagination is a useful attribute in evaluating the outcome of biomedical innovation, the next step is to reflect on these future outcomes. To do this, students have to evaluate and rate certain outcomes on their desirability and make judgments based on this information. The final step after making judgments regarding the desirability of research outcomes is thinking out ways to anticipate undesirable research outcomes. For this, students need to take the many uncertainties and extensive possibilities into consideration. This asks for students to create their own scenarios and find creative ways to circumvent undesirable ethical and societal implications.

#### Example of Teaching for Anticipation

One way of using scenarios in an educational setting and helping students consider both the hard and soft impacts of research processes is to stimulate reflection on pre-built scenarios. By using narratives that show the dilemmas that can emerge in a research process and the consequences of particular decisions, students can see which key steps lead to specific outcomes and reflect on the desirability of these outcomes. These pre-built scenarios can be specifically developed for educational purposes, as well as artistic works like science fiction. Written narratives and movie scripts not only stimulate moral imagination, but can additionally help students see how the writers, who often do not have a scientific background, view certain research advances (Burton et al., 2018). A relevant scenario to stimulate competencies of anticipation in biomedical students may concern a technology that potentially has controversial applications. This can concern the potential of dual use of the technology, meaning that a technology intended for human benefit can intentionally be misused for harmful purposes, or a technology in which unintended negative outcomes lead to discussion. When considering a technology with controversial applications, it is helpful to reflect on historical cases that previously have led to large controversies. Students can use these historical examples to reflect on the steps that were taken to prevent negative side effects of the technology and explore whether this method of anticipation might also work for the scenario they are considering, or whether other methods might help to anticipate unintended outcomes. Students can practice distinguishing between hard and soft impact by describing all the outcomes they connect to a specific innovation. When asked to name potential outcomes of new technologies, students will probably start by naming hard impacts and have difficulty recognizing the soft impacts of the technology. Asking students to discuss how society might change when these new technologies are used for various applications will help them recognize the soft impact. To test if they thoroughly developed the competency to distinguish between hard and soft impacts, and can transfer this onto new examples, students can be asked to include hard and soft impacts in an essay or a scenario written by the students themselves.

### Reflexivity

The second dimension described by Stilgoe and colleagues is reflexivity, which is described as reflecting on one’s activities and commitments, understanding the limits of scientific knowledge, and becoming aware that a specific framing of an issue might not be universal (Stilgoe et al., 2013). When unpacking this description of reflexivity, it can both be interpreted as a call for epistemological reflection and a call for moral reflection. For biomedical scientists, reflexivity on both epistemological and moral questions is valuable when striving for responsible conduct of research. Epistemological questions lead to a reflection on why we know things and what justifies claims of knowledge. For biomedical scientists, these questions include: What is the research question being answered? Are the appropriate methods used and what is the justification for these methods? What are the criteria for confirming or denouncing a hypothesis? What conclusions can be drawn after the research is completed? Answering these types of questions might help researchers be more thoughtful in the communication of their research results and more transparent about uncertainties. When reflecting on ethical dimensions biomedical scientists should consider questions such as: Who benefits from certain research outcomes? Who might be negatively impacted by the research? Is there an appropriate informed consent procedure? Is the use of animal models appropriate in this type of research? Consideration of these types of questions might stimulate researchers to consult with ethicists or the public to make sure research outcomes are aligned with societal values.

The competencies described by Tassone and colleagues that are congruent with reflexivity are self-awareness, situational awareness, social awareness or empathy, ethical thinking, and disruptive thinking (Tassone et al., 2017). In these competencies, we recognize the importance of self-reflection, as well as reflection on the biomedical field as a whole, meaning that educational efforts for students should stimulate both a critical reflection on their own work, as well as work from others. Additionally, knowledge of what the guidelines of scientific integrity entail and what normative decisions biomedical researchers make in their daily practice can prepare students for the difficult choices they might face in the future. Disruptive thinking can be translated into the skill to think outside set boundaries, but also have the courage to speak up when one feels errors have been made or rules of integrity have been broken. Although the importance of an epistemological reflection is not specifically mentioned in these competencies, biomedical scientists need the skills to reflect on how science produces new knowledge and understand the limits of this knowledge. Therefore, we continue by describing learning goals and educational methods that can be valuable in stimulating this epistemological reflection. Subsequently, we describe how the framework of ethical decision-making can inform learning goals and instructional methods that stimulate reflection on scientific integrity and moral responsibilities.

#### Epistemological Reflexivity

In biomedical education, there is a strong focus on the knowledge that is produced in research efforts and the practical skills researchers need when working in a laboratory. However, to understand the role research has in producing knowledge and the potential pitfalls of research methods, it is important that researchers also reflect on the epistemological foundations of their field. This form of meta-research finds its origin in the philosophy of science, and aims to evaluate and improve research practices by looking at the used methods, reporting, and reproducibility of research (Ioannidis et al., 2015). To participate in this form of reflection, it is important to teach students research methodology and specifically address the justification of the methods used (Grüne-Yanoff, 2014). Additionally, students should learn reasoning skills, and how to avoid reasoning fallacies. By teaching students to recognize faulty reasoning, like inferring positive conclusions from negative data, false generalization, or confusion between statistical correlation and causality, they learn to be more thoughtful when choosing methodologies and careful in drawing preliminary or faulty conclusions (Boniolo & Campaner, 2019). This asks for students to not only understand how the scientific process works, but also for students to recognize their own role in the production of new knowledge with its limitations and uncertainties.

To practice epistemological reflexivity, it is important that students reflect on their own work, as well as the work of others. This can be done in teaching activities in which the work of senior scientists is critically analyzed. Showing students that even papers published in prominent journals can contain faulty lines of reasoning or draw overly optimistic conclusions, helps them see the importance of properly reflecting on research methods, valuing peer review processes, and remaining critical even after scientific knowledge has been published. Additionally, criticizing and being criticized by fellow students is an important vehicle for stimulating reflexivity. Specifically addressing how to write the methodology section of papers and having students explain or reproduce research based on its methodology could show them the importance of reproducibility and stimulate them to be attentive when writing the methodology of a research paper.

#### Scientific Integrity and Moral Reflexivity

Reflecting on the moral responsibilities associated with being a researcher is a fundamental competency for participating in responsible research. Moral responsibility entails among other things, adherence to the principles of transparency and integrity, including avoiding fraud and scientific misconduct, sharing important results with others, being open to criticism, and serving as a peer reviewer for fellow scientists (Douglas, 2003). It is important that students understand these responsibilities and recognize their importance.

Biomedical scientists often participate in research that uses animals, which brings additional ethical questions but also rules and legislation that biomedical students have to understand. The same has to be argued for research using human biospecimen and research involving human trial participants. We do not argue that it is necessary for undergraduate biomedical students to be able to recall all the rules regarding experimentation on human or animal subjects. We believe this could better be approached as an affective goal in which students learn to value and respect the rules concerning research involving human subjects or animals. To achieve this, not only a reflection on current practices (e.g., ethical reflection on clinical trials and the use and sharing of personal data) but also a historical reflection (e.g., concerning the Tuskegee Syphilis Study, or the origin of the first immortalized human cell line) is important. This historical reflection can help to show how these examples of unethical research have led to international research ethics codes like the Belmont Report and the declaration of Helsinki that have a strong influence on current practice.

When considering the ethical aspects of biomedical research, it is insufficient to have scientists solely rely on personal judgment without any previous training on how to reflect on the ethical dilemmas that might arise. To help future scientists make these normative considerations, they need certain ethical competencies. A framework considering the skills necessary for ethical decision-making is described by Rest and colleagues (Rest, 1986). In this framework, the process of ethical decision-making is divided into four fundamental steps: ethical sensitivity, ethical reasoning, ethical motivation, and ethical action. The first two steps in this process (ethical sensitivity and ethical reasoning) can be recognized in the competencies of reflexivity described by Tassone and colleagues and serve as learning goals for education for moral reflexivity. The latter two steps can be recognized in the dimension of responsiveness and are described later in this paper. Ethical sensitivity is the skill of identifying ethical aspects of a situation, including an intuition regarding others’ comfort and well-being. Ethical reasoning is the ability to reason about the ethical aspects of a dilemma and make judgments about which course of action is morally right.

#### Example of Teaching for Reflexivity

Reflexivity can be practiced by looking at the epistemological, methodological, ethical, and/or societal dimensions of different case studies. These can either be real life or imaginary but plausible cases. For the epistemological and methodological cases, students can start by looking at the abstract of research papers. When looking at these abstracts, they should focus on the foundational steps in a research process, for example. distinguishing the research question that is being answered, why the specific methodology was used, what other methodologies could be used, or if the described conclusions are plausible and not overly optimistic.

When reflecting on the ethical aspects of using animals and human subjects, students can discuss unethical or dubious examples. Formulating their own rules on how to avoid this unethical research helps them to learn to value rules regarding the use of animals and human subjects in research. By comparing their own rules to the established rules, for example, described in the declaration of Helsinki, they’ll recognize that they might have overlooked some ethical aspects or aspects in which they were more strict than the set rules. To train the skill of ethical sensitivity as part of moral reflexivity, the four principles of bioethics described by Beauchamp and Childress can serve as the starting point (Beauchamp & Childress, 2001). By examining the principles of autonomy, beneficence, non-maleficence, and justice in case studies and narratives based on real-life situations, students see that ethical principles play a role in many biomedical research projects. Additionally, for expanding the skill of ethical sensitivity, teaching approaches can include discussions about the assumptions and values that accompany these principles, and how different values may cause conflicts in the described situation. To transition to ethical reasoning, students should think critically about the ethically justifiable course of action and discuss this course of action with others. Discussions with fellow students about why they believe this course of action is morally right shows them that people can have different opinions about the values that should be deemed most important. This is an essential insight for realizing that many different perspectives must be considered in an ethical dilemma.

### Inclusivity

The third dimension described by Stilgoe and colleagues is inclusivity, which stresses the importance of inclusion of additional voices in making decisions concerning developments in research and innovation. These voices not only include experts in different (scientific) disciplines but also stakeholders, and members of the public. They should be involved in a dialogue that takes place early in the scientific process (Stilgoe et al., 2013). Stakeholders in biomedical research range from patients who potentially benefit from the new research to pharmaceutical companies and clinicians involved in the development and clinical implementations of biomedical technologies. Additionally, development of new technologies that can fundamentally change society requires early involvement of policymakers and the public in discussing the desirable outcomes of these developments. Different disciplinary experts who should be involved in multi-disciplinary discussions concerning biomedical innovation include ethicists, social scientists, and legal experts. Inclusivity of these different voices leads to higher legitimacy of research efforts and could contribute to greater trust of science in society.

Tassone and colleagues connect four competencies to the dimension of inclusivity: trans-disciplinary collaboration, participatory ability, multi-perspective and intercultural communication, and openness and transparency (Tassone et al., 2017). Reflecting on this, the described competencies can be seen as two-fold: the ability to communicate and to collaborate with the different groups affected by the research. Although Tassone and colleagues mention trans-disciplinary collaboration as one of the competencies for inclusivity, we feel that the term “multi-disciplinary collaboration” is more appropriate for describing this collaboration between different disciplines, because this collaboration does not necessarily transgress the borders of separate disciplines. In addition to communication and collaboration skills, future scientists have to recognize that research can benefit from inclusion of different perspectives. These include disciplinary perspectives, as well as non-scientific perspectives, such as emotional or cultural perspectives. Becoming aware of the value of these different voices is, therefore, an additional goal for education aiming for inclusivity. In the following section, we describe learning goals and teaching activities that concern learning to value different perspectives, as well as skills for communicating and collaborating with experts from different disciplinary backgrounds, societal stakeholders, and the public.

#### Value Different Disciplinary Perspectives and Multi-Disciplinary Collaboration

To participate in multi-disciplinary collaborations, contributors must understand how different perspectives guide varied ways of thinking (Boon et al., 2019). These perspectives cause experts from different disciplines to ask different questions, refer to different discipline-specific knowledge, and use diverse methods and technologies to come up with solutions. Biomedical students have their own disciplinary perspective that is implicitly transferred to them by the biomedical researchers who educate them. Students should become aware of their disciplinary perspective and the potential biases it can cause. Furthermore, biomedical students should learn to appreciate that people with different backgrounds have different perspectives, and their views are valuable in multi-disciplinary collaborations. To make sure that students are aware of the insights and perspectives of various academic disciplines, it is important that they encounter professionals from these different disciplines in their education. An often-suggested method for doing so is to let students contribute to a multi-disciplinary project with students from other disciplines. However, education in multi-disciplinary groups might not be an effective teaching strategy if students are not prepared to communicate and collaborate with students from other disciplinary perspectives. As a starting point for teaching these communication and collaboration skills, students need to understand the epistemological foundations of other disciplines and how knowledge is constructed in those disciplines (Boon & Van Baalen, 2019). A useful approach to learning the epistemological foundations of other disciplines is to have teachers with different disciplinary backgrounds teach about topics that are relevant for biomedical scientists. Seeing how different experts reflect on a biomedical subject from their disciplinary perspective, and the methodologies they use to answer questions, might help students appreciate these different perspectives. Another valuable intervention might be to ask students to put themselves in the shoes of an expert from another field and argue from another disciplinary perspective. This form of perspective-taking should not be done with the goal of becoming an expert in other disciplines, nor to learn how to reason exactly like the disciplinary expert, but it can help students realize potential bias in their disciplinary perspective and understand that other, perhaps better, methods to examine certain questions and problems can be found in other disciplines.

#### Value Non-scientific Forms of Knowledge, Emotional Perspectives, and Bi-directional Communication

In addition to recognizing perspectives from different scientific disciplines, students should also recognize the added value of non-scientific forms of knowledge, such as experiential and cultural knowledge. As science does not operate in a vacuum, society should be involved in the production and evaluation of new scientific knowledge (Jasanoff, 2004). In particular, research that concerns new treatment methods should include patients’ experience to increase the relevance and quality of the research (Caron-Flinterman et al., 2005). In like manner, emotional perspectives are to be considered when discussing developments in biomedical research. These perspectives, shaped by local, cultural, and social norms, determine how the public feels towards biomedical research and the innovations that result from the research. Consequently, biomedical sciences students need to learn how to interact with the public, when considering the ethical and societal impact of their field. For this interaction to become truly inclusive of public perspectives, it is not sufficient to use a unidirectional approach to science communication in which the public is considered as a passive recipient of knowledge (Dudo & Besley, 2016; Nisbet & Scheufele, 2009) Additionally, it should be warranted that rather than thinking *for* the public, researchers should engage *with* the public. When efforts to engage with the public are not considered carefully, they might endorse stereotypes and reinforce the bias of biomedical researchers, rather than stimulating inclusivity (Warren et al., 2018). Therefore, preparing students to participate in a dialogue or bi-directional form of communication, in which there is a two-way flow of information from the expert to the public and vice versa, is an essential part of a biomedical curriculum.

Work by Reincke et al. describes three recommendations for scientists that interact with the public using such a dialogue approach (Reincke et al., 2020). These recommendations can be used in defining learning goals that prepare for participating in bi-directional communication with the public. The first recommendation proposed by Reincke and colleagues concerns the responsibility of experts to share knowledge in an accessible way, congruent with the needs of conversation partners. Discussing new scientific innovations in a manner that is beneficial for both scientists and the public requires all participants to be able to recognize the meaning of innovations in their personal lives, as well as for society at large. A congruent learning goal for biomedical students is the ability to discuss science in a way that is comprehensible and relevant for people of different cultural backgrounds and levels of scientific knowledge. This means that students should be able to communicate scientific ideas without using discipline-specific jargon, with a focus on meaning instead of technical details. The second recommendation for experts is to be open to listen and learn from the public, meaning that research should be responsive to multiple perspectives. For students, this necessity to listen and learn to the public translates into recognizing and valuing non-scientific knowledge and emotional perspectives. The final recommendation is to invest in relations with conversation partners. This relationship should be based on a mutual feeling of trust. To be able to create a relationship based on trust with the public, students need to learn to be open in transparent in their communication. This includes sensitive communication about uncertainties and risks, and honesty about the limits of knowledge.

#### Example of Teaching for Inclusivity

An example of how students can learn to appreciate inclusivity is by allowing them to actively participate in, and reflect on, societal dialogues. In such dialogues, experts from different disciplinary backgrounds, stakeholders, and members from the public discuss the desirability of scientific innovations. Reflecting on the role the experts take in public dialogues can help them to recognize their own responsibilities in interaction with the public. Furthermore, students can recognize the role of different disciplinary perspectives in discussing the outcomes of biomedical innovations. Comparing their own views and opinions with those of other disciplinary experts, stakeholders, and the public will help them recognize their own disciplinary perspective and appreciate that people from different backgrounds view things from a different perspective. Having students facilitate and participate in actual ongoing societal dialogues can be regarded as a form of community-engaged learning. By taking the role of an expert in their own dialogue sessions, students can practice their science communication skills. By involving members of the public, students get real experience with open and transparent communication as well as translating biomedical knowledge to information that is comprehensible for a layperson audience. Additionally, by engaging in such a dialogue with a layperson audience, students can become aware of the contributive ideas the public might have to help the progress of biomedical research.

### Responsiveness

The fourth and last dimension described by Stilgoe and colleagues is responsiveness. For responsible conduct of research, it is not only important to ask the right questions but also respond to these questions and use new information to change the course of action (Stilgoe et al., 2013). Although responsiveness is an obvious last addition to the dimensions of responsible research and can be seen as the final objective, it is the most difficult one to translate into a specific skill set. An example of responsivess in biomedical research is a researcher who shows responsibility towards the ethical and societal dimensions of his research and is willing to drastically change the direction of a research project based on criticism from outside voices, even if this stalls the research process. However, this requires a specific attitude rather than a specific set of skills that can be practiced. When reflecting on the competencies of responsiveness described by Tassone et al., navigating complexity/wickedness, adaptability, and agency, we recognize these behavioral goals in adaptability and agency. Because biomedical students, especially at an undergraduate level, do not have the real-life experience of being responsible for the course of action in large research projects, they have difficulty imagining the challenge of changing their research direction once it is set in motion. Additionally, it is impossible to ensure that students translate attitudes taught in an educational setting into their future research practice. It might, therefore, be unrealistic to expect students to master the competencies of agency and adaptability after a few years of education. However, when aiming for these competencies of responsiveness, the first step is to show the complexity and wickedness of the issues that they will face in their career. Awareness of this complexity, together with the skills described previously that are congruent with the dimensions of anticipation, reflexivity, and inclusion, might serve as the first stepping stone to help students develop the attitudinal aspects necessary for agency and adaptability. In the following paragraphs, we further explain this line of reasoning.

#### Teaching for Complexity and Wickedness

As society is developing, many of the problems we face are becoming more complex. These complex, or wicked, problems have certain characteristics, including that there is seldom a single right answer, and attempts to a solution may have unintended consequences that could cause new problems (Rittel & Webber, 1973). Additionally, wicked problems often cannot be solved within the boundaries of a single discipline. Teaching students to think in an interdisciplinary way can help them navigate this complexity. In contrast to multi-disciplinary competence, which focuses on working together with experts from different disciplines, interdisciplinary competence asks for a transcendence of the border of a single expertise and combines knowledge from different disciplines to attain new ideas. To learn to bridge different disciplines, reflection on an issue from various angles has to be stimulated (Boon & Van Baalen, 2019; Grüne-Yanoff, 2014; Spelt et al., 2009). This can be done by reflecting on case studies and current world problems in which this complexity, and therefore the necessity for an interdisciplinary approach, can be recognized. In these wicked problems, biomedical research can be the cause of the problem, the solution to the problem, or even both. Recognizing the role of biomedical sciences in both creating and solving these wicked problems can be the first stepping stone to become responsive. By looking at role models who contribute to solving wicked problems with an interdisciplinary approach, students can understand how scientists can be responsive. The focus of these efforts should be how certain responses shaped society and which additional problems arose when choosing this course of action, showing that there is not a perfect response to complex and wicked problems.

#### Preparing for Agency and Adaptability

The next step in responsible conduct of research is agency, followed by adaptability. Agency can be described as the competency and desire to practice responsible research. In the earlier mentioned framework of ethical decision-making, this translates to the steps of ethical intent. In the same framework for ethical decision-making, adaptability can be recognized as ethical action, the step in which ethical intent translates into a corresponding action (Rest, 1986). These competencies can be seen as the ultimate goal for education in responsible conduct of research. However, teaching specific intentions and actions might not be an effective approach, as there is a disparity between knowing and following the right course of action (Clarkeburn, 2002). Without real-life experiences, students have difficulty understanding how external pressures caused by lack of funding, publication indexes, and media coverage, for example, might influence their actions. This makes it difficult to ensure that educational approaches truly lead to certain ways of acting in the future. However, educational approaches can stimulate the competencies students need to strengthen their motivation for moral agency and adaptability. Many of the learning goals described in the previous sections focus on seeing the value of efforts that promote anticipation, reflexivity, and inclusivity. By creating awareness of the potential value of these dimensions, students might become more motivated to participate in these types of efforts. According to the theory of self-determination, motivation is mediated by a perception of competence (Ryan & Deci, 2000). It is presumed that the educational efforts described in this paper will build the student’s confidence and competence. Therefore, making these learning goals an integrated part of biomedical curricula prepares students to become responsive biomedical researchers who participate in responsible biomedical research efforts. The first steps towards becoming a responsive professional can already be taken in biomedical training, most effectively in educational settings that resemble the future working environment as closely as possible, for example, during internships, interdisciplinary group projects or community-engaged learning.

#### Example of Teaching for Responsiveness

Biomedical research can be both part of the cause and the solution to a wicked problem. The current Covid-19 pandemic can be seen as an example of such a wicked problem. By discussing different aspects of a wicked problem students can appreciate that taking responsive actions, when dealing with wicked problems helps to find better solutions. As an exercise, students can think out the process of vaccine development during a worldwide pandemic. As the urgency for a vaccine, during a pandemic is high, students have to think about ways to speed up the process of vaccine development, considering rules of safety and ethical guidelines. Additionally, the need for worldwide distribution of the vaccine asks for a deliberation that involves many different stakeholders to determine how to develop, test, and distribute the vaccine. Looking at this case, it becomes clear that vaccine development in case of a pandemic involves many different facets that ask for an interdisciplinary and responsive approach.

## Curriculum Embedment

When responsiveness to the ethical and societal dimensions of the biomedical research field is regarded a crucial learning objective in biomedical education, educational activities that stimulate anticipation, reflexivity, inclusivity, and responsiveness should have a designated place in biomedical educational programs. However, proper implementation of all the suggested learning goals can only be achieved with extensive curriculum reform. Although it is beyond the scope of this paper to discuss in detail how such curriculum reform could be designed from the program down to the level of individual courses, we will elaborate on some of the practicalities of implementation that are worthy of consideration.

The first consideration is the strategy for implementation. Two distinctive strategies have been suggested by others. The first is the implementation of dedicated modules or courses in which the ethical and societal dimensions are discussed extensively (Bryant & Baggott La Velle, 2003). The second approach is a more spread-out approach in which the societal and ethical aspects are discussed across the curriculum, and as an integral part of already existing courses (Cech, 2015; Wimmers & Gasparich, 2014). In this second approach, educational activities that stimulate reflection on the ethical and societal dimensions can be linked to the content knowledge that is taught at specific moments. This means, for example, that an introductory course in genomics can discuss the importance of public engagement in deciding whether we should use new technologies to edit the DNA of embryos, or that a course in pharmacology can discuss the ethical and legal consideration of doing a drug trial in low- and middle-income countries. However, solely using this rather fragmented approach might not be sufficient if we want students to truly master the described learning goals. An approach in which both dedicated courses and spread-out learning activities are combined, might therefore be most favorable. In a dedicated and obligatory course, students can develop the foundational competencies for analyzing the ethical and societal dimensions in their field. These competencies can then be further developed and applied in a more practical context via short modules or sessions in existing courses in the curriculum.

A second consideration concerns who should teach biomedical students about the ethical and societal dimensions of their field. One approach is inviting disciplinary specialists (e.g., philosophers, ethicists, or social scientists) to teach these students. Another approach is to have biomedical scientists teach these aspects of the biomedical field. However, as both the disciplinary specialists and the biomedical scientists are specialists in their own field, their disciplinary perspectives might be a limitation in teaching students to see the diverse perspectives that are relevant when discussing the ethical and societal dimension of biomedical research. Multi-disciplinary collaborations between these educators in shaping, and developing educational material, and teaching these ethical and societal dimensions might therefore be an interesting approach. In such a co-teaching construction, teachers from different backgrounds can show that biomedical topics can be viewed from different perspectives. At the same time, they can serve as role models in showing students the benefits of multi-disciplinary collaboration.

The final consideration for curriculum embedment of the suggested learning goals is developing valid tools for assessment. Ethical competencies are often assessed by using open ended-questions or grading essays written by students. However, when trying to assess if students have truly mastered and internalized the competencies to conduct responsible biomedical research, such assessment methods might not be sufficient. Useful approaches can be found in teacher education in which methods are described to assess teacher dispositions (Burant et al., 2007; Schussler et al., 2010). Another approach that might be applicable for assessing the competencies to reflect on the ethical and societal dimensions of biomedical research might be the framework of “entrustable professional activities” (EPAs) which is developed for medical education (Ten Cate & Scheele, 2007). In this approach,competencies are assessed based on the level of responsibility that a teacher believes can be entrusted to the student.

## Concluding Remarks

In this paper, we analyzed how the policy framework of Responsible Research and Innovation can inform teachers about what future biomedical scientists need to know and do in order to contribute to responsible research practices. We analyzed the four dimensions defined by Stilgoe and colleagues (anticipation, reflexivity, inclusivity, and responsiveness) in relation to ideas and frameworks from other disciplines, to define learning goals and outline teaching approaches for biomedical students. A summary of the proposed learning goals can be found in Table 1. We found that the proposed dimensions can be worked out into concrete learning goals and congruent teaching activities. Regarding the final dimension, responsiveness, we propose that learning activities that stimulate both *seeing the value of* and *building a feeling of competence in* the dimensions of anticipation, reflexivity, and inclusivity will lay the foundations to become responsive biomedical scientists and, therefore, responsible societal actors in the future.

Reflecting on what science is, what it ought to be, and what role scientists have played and should play in society, is what characterizes the work of scholars in the fields of history, philosophy, and sociology of science. Therefore, their work can be inspirational for the operationalization of the proposed learning goals. Many others have described the usefulness of insights and educational approaches from the disciplines of history, philosophy, and sociology of science. It is even suggested that students in the foundational fields of history, philosophy, and sociology have a better understanding of the nature of science than students in scientific disciplines (Akgun & Kaya, 2020). Nonetheless, we feel that many disciplinary programs in higher education barely make use of what these fields have to offer to the education of their students. However, simply implementing knowledge and course material from these specific fields into science curricula might not be sufficient. In their study assessing the impact of a history of science course for science students, Abd-El-Khalick and Lederman have suggested that to improve students’ understanding of the nature of science, educational approaches should focus on the conceptual framework of history of science (Abd-El-Khalick & Lederman, 2000). Using this approach, science education from a historical perspective can focus on the social, cultural, and political contexts certain biomedical discoveries were done. This can provide insights into the importance of today’s social-cultural context for scientific research (Jardim et al., 2021). From a philosophical perspective, this can entail critical reflection on how personal values influence the professional activities of scientists, and therefore how individual values in the future might shape their own research practices (Koster & de Regt, 2020). Science teaching from a sociological perspective can show the human side of science, by focusing on behaviors, values, practices, and attitudes of both scientists and society in accepting scientific knowledge (Allchin, 2004; Dunlop & Veneu, 2019).

The challenge that remains for scholars in science education lies in identifying design principles for concrete learning and teaching strategies that are well-grounded in academic insights derived from these fields. The scholarly approach to arrive at these design principles is educational design research (Akker et al., 2013; McKenney & Reeves, 2014). Our findings summarized in Table 1 can be the starting point for such design-based educational research to gain insight is the inner working of learning and teaching activities that aim for developing competencies in anticipation, reflexivity, inclusivity, and responsiveness.

This paper is specifically written with a focus on biomedical scientists, which means that we seek to identify learning goals that are in a way domain-specific for this group. Although, competencies on these four dimensions are relevant for students in all fields, we believe that expertise in responsible research processes should be stimulated in close connection with the *disciplinary* role that students will have in these processes. Therefore, a domain-specific interpretation and elaboration of the four dimensions into concrete learning goals and activities is essential. We hope that our analysis inspires educators in other disciplines to make comparable analyses for the needs of their students. We feel that in this way we can all take our responsibility as discipline-based educators to help our students in becoming broadly oriented, responsive actors in our changing society.
